# Particulate matter from car exhaust alters function of human iPSC-derived microglia

**DOI:** 10.1186/s12989-024-00564-y

**Published:** 2024-02-15

**Authors:** Henna Jäntti, Steffi Jonk, Mireia Gómez Budia, Sohvi Ohtonen, Ilkka Fagerlund, Mohammad Feroze Fazaludeen, Päivi Aakko-Saksa, Alice Pebay, Šárka Lehtonen, Jari Koistinaho, Katja M. Kanninen, Pasi I. Jalava, Tarja Malm, Paula Korhonen

**Affiliations:** 1 A.I. Virtanen Institute for Molecular Sciences, University of Eastern Finland, Kuopio, Finland; 2 F.M. Kirby Neurobiology Center, Boston Children’s Hospital, Boston, MA USA; 3 Stanley Center for Psychiatric Research, Broad Institute of MIT and Harvard, Cambridge, MA USA; 4 Division of Eye and Vision, Department of Clinical Neuroscience, St. Erik Eye Hospital, Karolinska Institutet, Stockholm, Sweden; 5 VTT Technical Research Centre of Finland, Helsinki, Finland; 6 Department of Surgery, Royal Melbourne Hospital, The University of Melbourne, Melbourne, VIC 3010 Australia; 7 Department of Anatomy and Neuroscience, The University of Melbourne, Melbourne, VIC 3010 Australia; 8 Neuroscience Center, University of Helsinki, Helsinki, Finland; 9 Department of Environmental and Biological Sciences, University of Eastern Finland, Kuopio, Finland

**Keywords:** Microglia, Particulate matter, Traffic-related, iPSC, iPSC-microglia, Neuroinflammation, Air pollution, Diesel, Glia, Human

## Abstract

**Background:**

Air pollution is recognized as an emerging environmental risk factor for neurological diseases. Large-scale epidemiological studies associate traffic-related particulate matter (PM) with impaired cognitive functions and increased incidence of neurodegenerative diseases such as Alzheimer’s disease. Inhaled components of PM may directly invade the brain via the olfactory route, or act through peripheral system responses resulting in inflammation and oxidative stress in the brain. Microglia are the immune cells of the brain implicated in the progression of neurodegenerative diseases. However, it remains unknown how PM affects live human microglia.

**Results:**

Here we show that two different PMs derived from exhausts of cars running on EN590 diesel or compressed natural gas (CNG) alter the function of human microglia-like cells in vitro. We exposed human induced pluripotent stem cell (iPSC)-derived microglia-like cells (iMGLs) to traffic related PMs and explored their functional responses. Lower concentrations of PMs ranging between 10 and 100 µg ml^−1^ increased microglial survival whereas higher concentrations became toxic over time. Both tested pollutants impaired microglial phagocytosis and increased secretion of a few proinflammatory cytokines with distinct patterns, compared to lipopolysaccharide induced responses. iMGLs showed pollutant dependent responses to production of reactive oxygen species (ROS) with CNG inducing and EN590 reducing ROS production.

**Conclusions:**

Our study indicates that traffic-related air pollutants alter the function of human microglia and warrant further studies to determine whether these changes contribute to adverse effects in the brain and on cognition over time. This study demonstrates human iPSC-microglia as a valuable tool to study functional microglial responses to environmental agents.

**Supplementary Information:**

The online version contains supplementary material available at 10.1186/s12989-024-00564-y.

## Background

Air pollution is almost impossible to avoid, contributing annually to 3.1 million deaths world-wide [1]. The effects of air pollution on respiratory system are well established with half a million lung cancer deaths and 1.6 million deaths caused by chronic obstructive pulmonary disease annually [2]. It also affects other systems within the body resulting in associations to cardiovascular disease, malignancies, diabetes, obesity, allergies, cognitive functioning and dementia [2]. A number of recent epidemiological studies link PM with increased risk of CNS disorders such as Alzheimer's disease (AD) [3–5], Parkinson's disease (PD) [6], stroke [7, 8], and dementia [9, 10]. Yet, the effects on the human brain have remained poorly understood due to the lack of suitable biological human research models since human brain cells have been difficult to obtain. With the invent of induced pluripotent stem cells (iPSCs), we now have unlimited source of different human cell types.

Traffic related particulate matter (PM) is the major source of outdoor air pollution particularly in highly populated areas [11]. PM is classified by size with diameters of 10.0, 2.5 and 0.1 µM corresponding to PM_10_, PM_2.5_ and PM_0.1_ respectively [12]. The coarse fraction (PM10–2.5) of PM is trapped in the airways while smaller ultra-fine particles (UFP) have a potential to passage through barriers and distribute throughout the body causing for example inflammation and oxidative stress in the tissues. There is a concern that UFPs could be more toxic, but this has still not been clearly shown. Furthermore, the fact that larger particles are “trapped” in the airways does not mean they are less harmful, or have less systemic effects [12, 13]. However, we are lacking comprehensive studies showing which PM components enter the human brain. Diesel exhaust (DE) is the primary component of traffic related PM, contributing especially to the smaller particles PM_2.5_ and UFP [14]. Diesel engines are the most prevalent traffic-related engines and are widely used in global industries including transportation, agriculture and construction [15, 16]. National regulations, standards and guidelines have been established to reduce traffic-related PM emissions. New diesel engines, diesel exhaust technology and biodiesels have been developed and studied, showing reduced carcinogenic risk and physiological effects [17]. Newer diesel technologies show smaller amounts of engine-out emission of PM, measured as reduced concentrations of non-volatile polycyclic aromatic hydrocarbon (PAH) and elemental carbon also referred to as black carbon [18]. EN590 is a current low sulphur emission standard for all automotive diesel fuels in the European Union. Based on sulphur content it has subclasses, Euro 1–6, standardized between 1993 and 2014. Compressed natural gas (CNG) fuel has a lower carbon content than diesel and it is considered to have less harmful emission because of its lead and sulphur free character. While diesel exhaust contains soot particles made up primarily of carbon, ash, metallic abrasion particles, sulphates and silicates, the PM emissions are typically low for CNG. Since CNG is gaseous fuel, its exhausts include primarily hydrocarbons, oxides of nitrogen (NOx), carbon monoxide (CO), carbon dioxide (CO_2_) and methane.

PM can cause brain effects mainly through two routes, either indirectly via circulating proinflammatory signals originating from peripheral tissues like lungs and liver [19], or by entering and accumulating directly in the brain via the olfactory route, especially in the case of the smaller-sized particles [20, 21]. In addition, soluble organic compounds, are considered highly important for systemic effects: they may cross rapidly into circulation without particle translocation. Also, translocation of particle-bound polycyclic aromatic hydrocarbons (PAHs) from the lung into the circulation far exceeds the translocation of UFPs. PM may also enter the brain from the circulation through a compromized blood–brain-barrier (BBB) or even induce microglial responses across an intact BBB, given their surveillance function [22]. Thus, PM may interact directly with brain immune cells, microglia, which largely orchestrate neuroinflammatory reactions in the brain [23].

Microglia are native immune cells of the brain with versatile functions. They contribute to blood–brain barrier integrity (BBB), clearance of apoptotic cells, and continuously survey, scan and react to any changes in the brain to protect neurons and maintain tissue homeostasis [24, 25]. Since microglia are highly dynamic cells and programmed to quickly react to environmental changes, exposure to PM can change their physiological properties and may contribute to pathological conditions [25–27]. Several mouse studies have shown that PM can induce neuroinflammation [28–30], microglial cytotoxicity and secretion of ROS [31, 32] possibly contributing to CNS damage, brain atrophy and cognitive deficits [33].

The studies using microglia obtained from animals or human cell lines may not inform whether and how live human microglia are affected by traffic related PM, since human microglia differ from mouse microglia both in their function and transcriptional signature [32, 34, 35]. We hypothesized that PM has an adverse effect on microglial functions, such as viability, metabolism and cytokine secretion. We also hypothesized that human iMGLs could be used for observing PM effects on microglial functions in vitro. While PM exposure has been studied using primary mouse microglia and immortalized microglial cell lines, such as BV2 cells, (as reviewed in [36]), we are lacking information how PM affects human microglia as they have been not accessible for research until recently upon development of stem cell derived iMGL models. Here, we describe the impact of two traffic-related pollutants, EN590 diesel and compressed natural gas (CNG) on the function of human induced pluripotent stem cell -derived microglia-like cells (iMGLs) in vitro. Our data show that microglia exhibit robust, yet pollution-dependent responses to EN590 and CNG exposures.

## Results

### Human iMGLs cluster around visible PM particles upon exposure

**Fig. 1:**
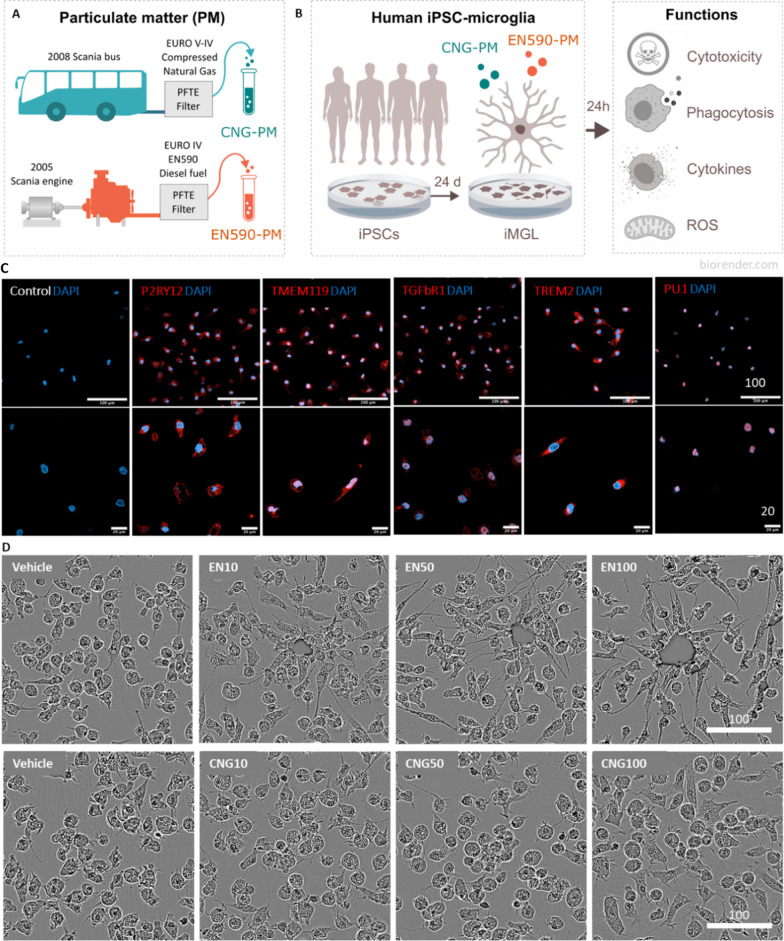
Study design and characterization of human iMGLs. **a** Schematic of collection of traffic-related air pollution PM samples. **b** Schematic of human iPSC-derived microglia-like cell (iMGL) cultures for functional assessment after 24-h PM exposure. Created partly with BioRender.com. **c** Immunostaining images of day 24 iMGLs at × 20 (top) and × 40 (bottom) magnification for control (only secondary antibody), P2RY12, TMEM119, TGFbR1, TREM2, and PU.1 antibodies in red and nuclei in blue. Scale bars 100 µm (top) and 20 µm (bottom). Cell lines: BIONi, TOB. **d** Representative phase contrast images of iMGLs treated for 24 h with vehicle (0.01% DMSO), 10, 50 or 100 µg ml^−1^ EN590-PM or CNG-PM. Scale bar 100 µm. Cell lines: BIONi, MBE, MAD6, TOB

We studied how human iPSC-derived microglia-like cells (iMGLs) react to 24-h exposure of traffic-related air pollutant PM collected under conditions simulating urban commuter buses driving multiple stops with low load [37] (Fig. 1a). EN590-PM was collected from the exhaust gas of conventional EN590 diesel fuel and CNG-PM from the exhaust of compressed natural gas (CNG) combusted in a 2008-year model commuter bus using a method that is widely used in the literature. The technical properties of PM were previously characterized and reported by VTT Technical Research Centre of Finland [37, 38]. A summary of chemical constituents (ng/mg PM mass) of EN590-PM and CNG-PM are presented in Table 1.

**Table 1:** Chemical constituents of PM emitted from the heavy-duty bus engine fueled with EN590 diesel and from a bus fueled with compressed natural gas (CNG) [37]

Composition	ng/mg PM		ng in 50 ug PM		ng per 96 well		fg/cell	
Organic constituents	EN590	CNG	EN59	CNG	EN59	CNG	EN590	CNG
PAH compounds	820	62	41	3.10	4.10	0.31	273	21
Genotoxic PAHs	380	26	19	1.30	1.90	0.13	127	9
Inorganic ions								
NO_3_^−^	1090	136	54.50	68.00	5.45	6.80	363	453
Na^+^	690	239	34.50	119.5	3.45	11.9	230	797
SO_4_^2−^	143	777	7.15	38.85	0.72	3.89	48	259
NH_4_^+^	137	199	6.85	9.95	0.69	1.00	46	66
Cl^−^	113	459	5.65	22.95	0.57	2.30	38	153
K^+^	24	116	1.20	5.80	0.12	0.58	8	39
Elemental composition								
Zn	2340	228	117.0	114.0	11.70	11.4	780	760
Fe	610	169	30.50	84.50	3.05	8.45	203	563
Cu	96	214	4.80	10.70	0.48	1.07	32	71
Mn	63	94	3.15	4.70	0.32	0.47	21	31
Cr	36	226	1.80	11.30	0.18	1.13	12	75
Co	24	313	1.20	15.65	0.12	1.57	8	104
Ni	3.6	267	0.18	13.35	0.02	1.34	1.20	89
Pb	3.1	20	0.16	1.00	0.02	0.10	1.03	6.67
V	1.8	27	0.09	1.35	0.01	0.14	0.60	9.00
Cd	0.2	1.1	0.01	0.06	0.00	0.01	0.07	0.37
Total PM burden	50 ug	50 ug	50 ug	50 ug	5 ug	5 ug	0.33 ng	0.33 ng

The amount of constituents in PM presented as ng/mg PM mass and amounts used for in vitro experiments serving as an example for the specific conditions per 50 ug of PM, per a 96 well and per an individual cell

*PAH* polycyclic aromatic hydrocarbon

Human iMGLs were differentiated from iPSCs obtained from four healthy donors whose characteristics are listed in Table 2 (Table 2, Fig. 1b). A previously published 24-day differentiation protocol was used to generate iMGLs that resemble human microglia both functionally and molecularly [39–41]. The microglial identity of iMGLs was confirmed using immunofluorescence staining of microglial markers [42–44] P2RY12 and TMEM119, TREM2, PU.1 and TGFbR1 (Fig. 1c). After 24-h exposure to EN590-PM, iMGLs clustered around the visible pollutant particles (Additional file 1: Video S1), whereas CNG-PM was not visible and did not cause morphological changes or cell clustering compared to vehicle treated iMGLs (Fig. 1d, Additional file 2: Video S2). Most likely these big agglomerates in EN590 sample are formed when organic fraction of diesel exhaust makes the particles to stick together. However, even though not visible in this image, EN590 sample contains also a very large number of single particles.

**Table 2:** Human iPSCs used in the presented work

Cell line	BIONi010-C-2	MBE2968 cl1	MAD6 cl 1	TOB064_F_B9_F3
Sex	M	F	M	M
Health status	Healthy	Healthy	Healthy	Healthy
Age, years	15–19	65	63	73
APOE type	ε3/ε3	ε3/ε3	ε3/ε3	ε3/ε3
Sample origin	Skin biopsy	Skin biopsy	Skin biopsy	Skin biopsy
Reprogramming method	Non-integrating episomal	Episomal nucleofection	Sendai virus	Episomal nucleofection
Karyotype	46XY	46XX	46XY	46XY
Reference	[42, 43]	[36]	[37]	[44]
Used in experiments				
ICC	Figure 1C	–	–	Figure 1C
Morphology	Figure 1D	Figure 1D	Figure 1D	Figure 1D
Cytotox	–	Figure 2A–D	Figure 2A, B, G	–
LDH	Figure 2E–J	–	–	–
MTT	Figure 2E–J	–	–	–
pHrodo	–	Figure 4	Figure 4	Figure 4
CellROX	Figure 6	Figure 6	Figure 6	–
Cytokine	–	Figure 7	Figure 7	Figure 7

### EN590-PM and CNG-PM do not affect cell viability or metabolism

**Fig. 2:**
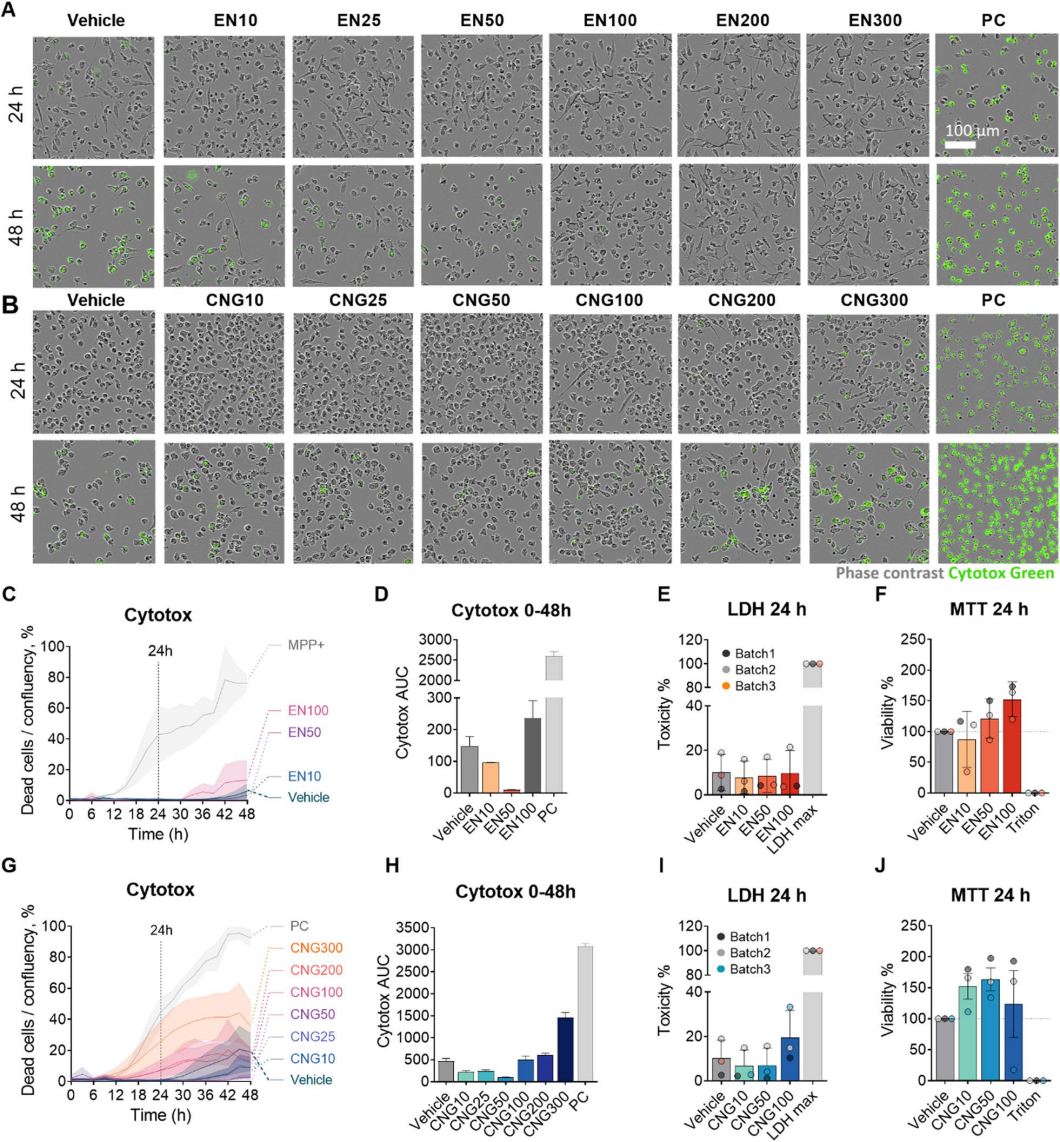
EN590-PM and CNG-PM are not cytotoxic. **a** and **b** Representative images of iMGLs treated with vehicle, 10–300 µg ml^−1^ EN-PM and CNG-PM, or positive control (PC: 400 µM 1-methyl-4-phenylpyridinium) at 24 h (top) and 48 h (bottom) with Cytotox Green Reagent labeling dead cells. Scale bar 100 µm. Cell lines: MBE, MAD6. **c** Time curve for Cytotox green positive dead cell count per cell density for vehicle and 10–100 µg ml^−1^ EN-PM treated iMGLs live-imaged for 48 h and normalized to PC at 48 h. Cell lines: MBE, MAD6. **d** Respective bar graphs for area under curves (AUC). Cell lines: MBE, MAD6. **e** Cell death assessed with the LDH test and **f** metabolic activity assessed with MTT test after 24-h exposure. Corresponding **g** Cytotox time-curve, **h** AUC, Cell lines: 2 × MBE, MAD6., **i** LDH and **j** MTT quantification for 10–300 µg ml^−1^ CNG-PM treated iMGLs. Cell lines: 3 × BIONi. **c** and **d** n = 2 experiments each with 3 wells. **e**–**j** n = 2–3 experiments each with 3–6 wells. Data as mean ± SEM. Non-parametric Kruskal–Wallis test followed by Dunn’s test. See also Fig. 3

**Fig. 3:**
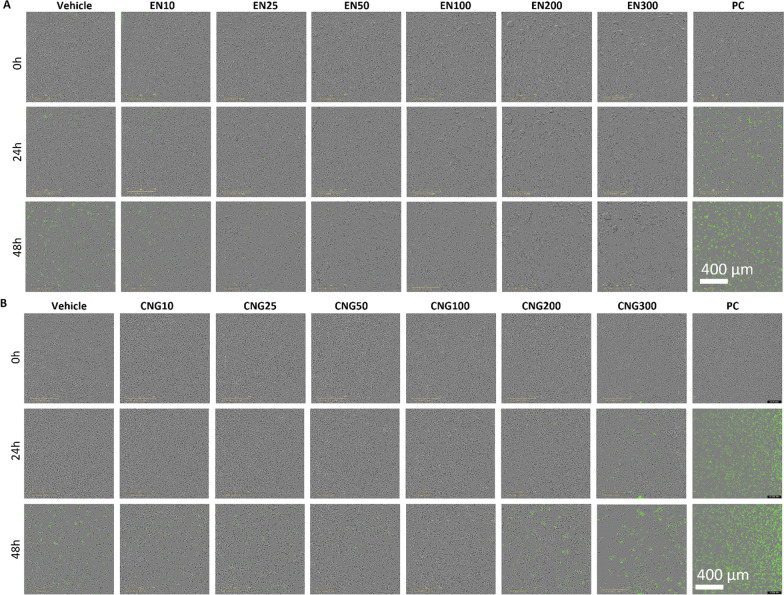
Representative images of iMGLs after 0, 24, or 24 h after treatment with vehicle or 10–300 µg ml^−1^ **a** EN-PM or **b** CNG-PM and Cytotox Green Reagent labeling dead cells. As a positive control (PC: 400 µM 1-methyl-4-phenylpyridinium) was used to kill cells over time. Scale bar 400 µm

Since the impact of EN590-PM and CNG-PM on viability of human microglia is not known, we first examined the cytotoxicity of these particles using Cytotox Green live-cell imaging assay. iMGLs were plated to experimental vessels on d16 of the protocol when they do not proliferate anymore and exposed to 10–300 μg ml^−1^ PMs, used in earlier in vitro studies [36] and shown to be cytotoxic for macrophages [37], and live-imaged for 48 h. Upon cell death, the cell membrane becomes compromised enabling Cytotox reagent to enter the cell and bind to DNA resulting in a green fluorescent signal. Bright field and fluorescent channels demonstrated little-to-no cell death in iMGLs exposed to smaller concentrations of EN590-PM (up to 100 µg ml^−1^) whereas vehicle and highest concentration of CNG-PM (300 µg ml^−1^) increased cell death over 48 h (Figs. 2a, b and 3). However, these differences did not reach statistical significance. The cells were not exposed to highest concentrations of EN590 (200 and 300 µg ml^−1^) due to poor availability of pollutant. Decreased viability of vehicle-treated cells can be explained by the lack of fresh media and cytokines. Time curves for Cytotox positive dead cells per cell density (Fig. 2c) and the respective area under curve (AUC) showed no significant differences between EN590-PM and vehicle (Fig. 2c, d). Quantification of Cytotox positive dead cells per cell density (Fig. 2d) and LDH test (Fig. 2e) at 24-h timepoint demonstrated no significant differences in permeability of cell membrane for viability indicators upon exposure to 10–100 µg ml^−1^ EN590-PM. MTT test, measuring mitochondrial activity of live cells, did not show significant differences in cell metabolism after 24-h exposure to EN590-PM either (Fig. 2f). CNG-PM showed strong trend to decrease cell death compared to the vehicle over 0–48 timeframe upon 50 µg ml^−1^ and 10 µg ml^−1^ exposures. However, these experiments were repeated only twice and thus, statistical significance could not be counted (Fig. 2g, h). Likewise, LDH assay (Fig. 2i) showed no compromised cell membrane, nor did MTT test indicate significant changes in metabolism at 24-h timepoint (Fig. 2j).

### EN590-PM and CNG-PM impair phagocytosis of iMGLs

**Fig. 4:**
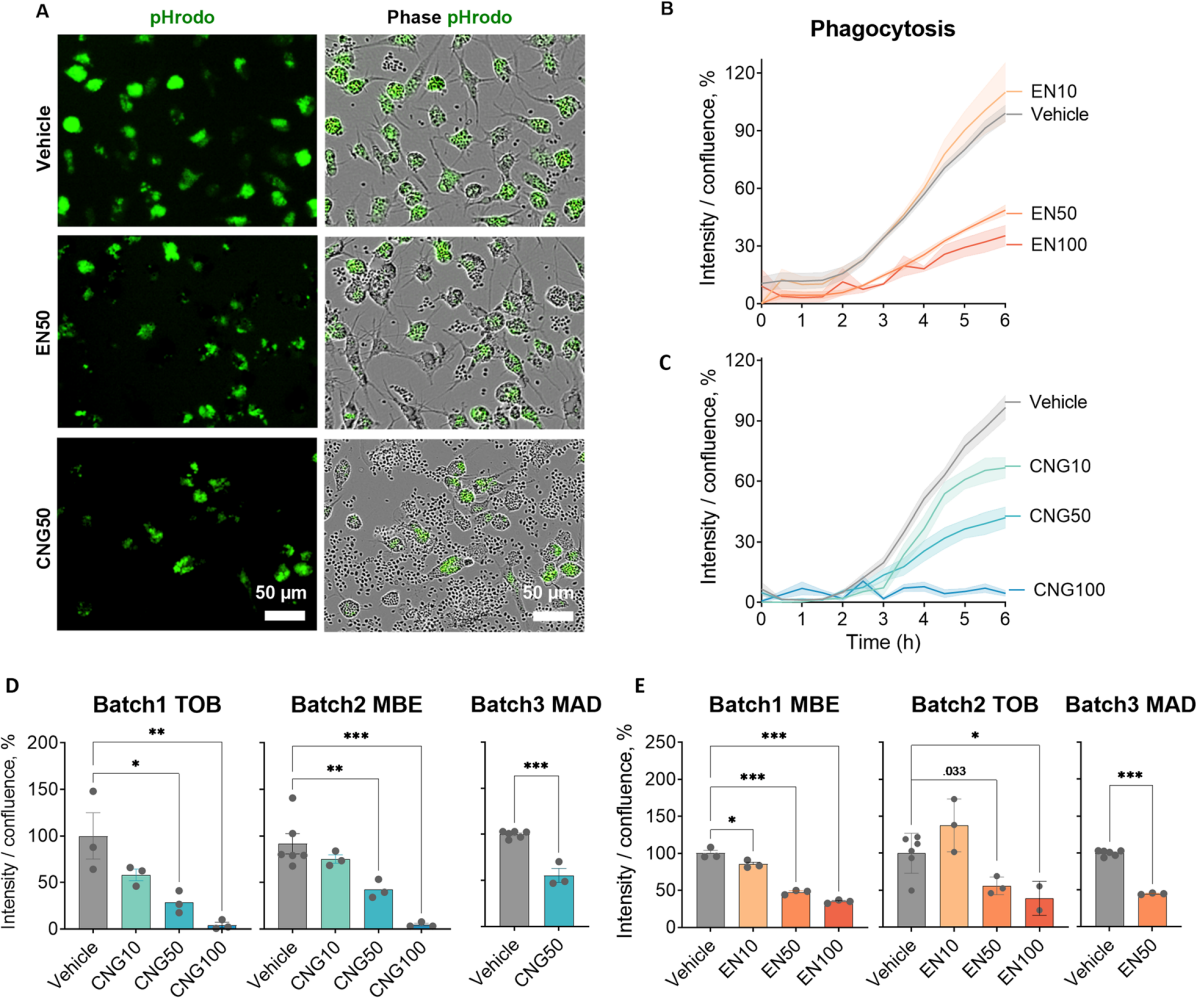
EN590- and CNG-PM impair phagocytosis of iMGLs. **a** Representative images for green fluorescent and phase contrast channels showing phagocytosed green pHrodo Zymosan A bioparticles with vehicle, 50 µg ml^−1^ EN590-PM and 50 µg CNG-PM treated cells at 6 h timepoint. Scale bars 50 µm. **b** Time curves for intensity of green fluorescent pHrodo bioparticles per cell density over 6-h live-cell imaging after 24-h exposure to 10, 50 and 100 µg ml-1 EN590-PM normalized to vehicle. **c** Time curves for 10, 50 and 100 µg ml^−1^ CNG-PM treated iMGLs **d** bar graphs for three independent iMGL batches exposed to 10, 50 and 100 µg ml^−1^ CNG-PM. **e** bar graphs for three independent iMGL batches exposed to 10, 50 and 100 µg ml^−1^ EN590-PM. Data as mean ± SEM. Each batch is analysed separately using One-way ANOVA. *p < 0.05, **p < 0.01, ***p < 0.001; n = 3–6 wells. Cell lines MBE, MAD6, TOB064

**Fig. 5:**
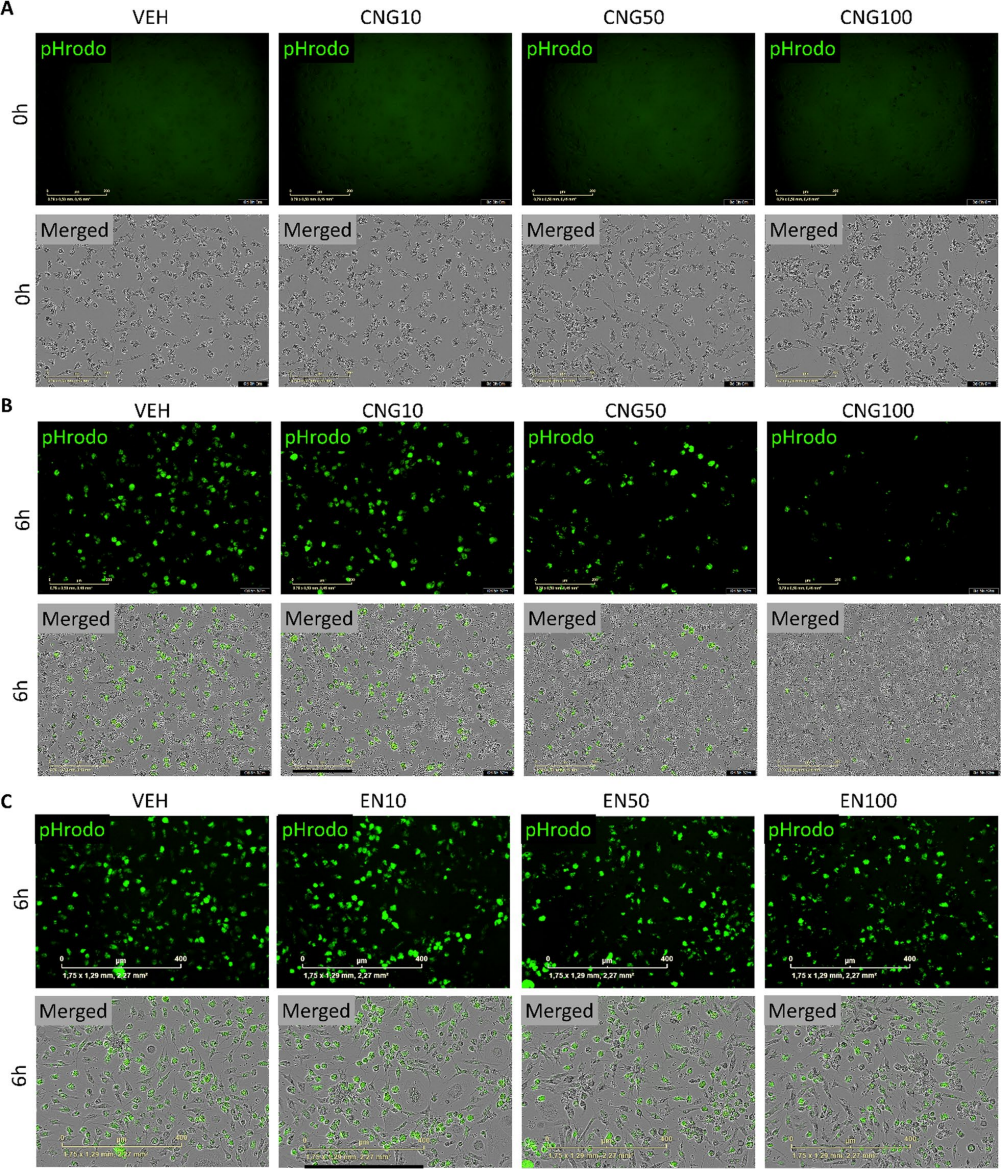
Representative images for phase contrast and green fluorescent channels showing phagocytosed green pHrodo Zymosan A bioparticles for vehicle or 10–100 µg ml^−1^ EN590-PM or CNG-PM treated cells at (**a**) 0 h timepoint and at (**b**, **c**) 6 h timepoint. Scale bars 200 µm for (**a**, **b**), 400 um for (**c**)

Since phagocytosis of cellular debris and pathogens is a key function of microglia within the CNS, we next examined how iMGLs phagocytose pHrodo labeled Zymosan A bioparticles under PM exposure using live-cell imaging (Figs. 4a and 5). Since 10–100 µg ml^−1^ doses of EN590-PM are used for rodent microglia studies [36], we investigated whether 10, 50 and 100 µg ml^−1^ exposures for 24 h would affect microglial phagocytosis. Indeed, 50 and 100 µg ml^−1^ EN590-PM impaired iMGL phagocytosis over the 6-h assay (EN50, *p* < 0.001; EN100, *p* < 0.001) (Fig. 4b, e). Similarly, CNG-PM reduced phagocytosis with 50 and 100 µg ml^−1^ concentrations (CNG50, *p* = 0.001; CNG100, *p* < 0.001) in a dose-dependent manner (Fig. 4c, d). Each batch is analysed separately.

### EN590- reduced and CNG-PM increased intracellular ROS production in iMGLs

**Fig. 6:**
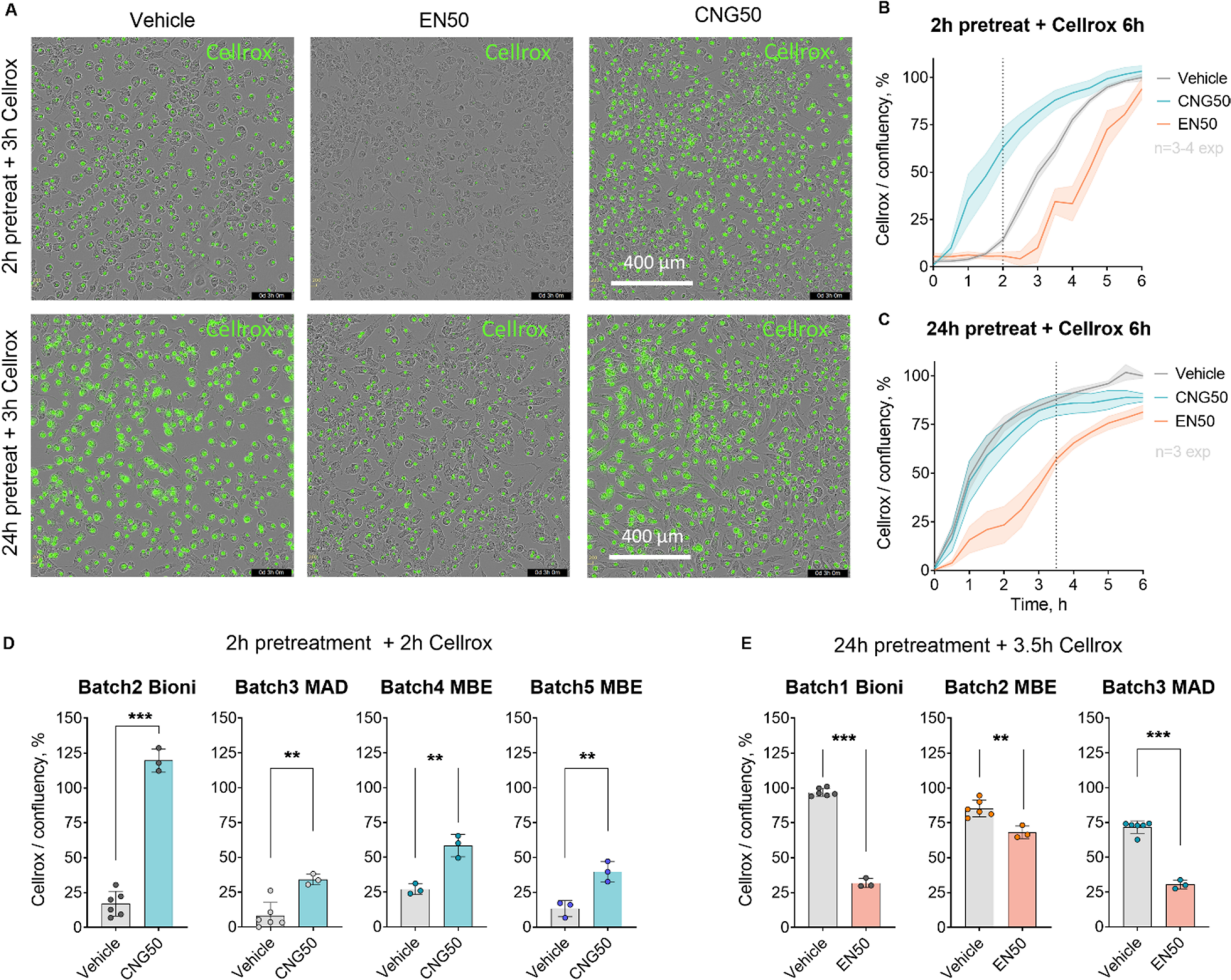
EN590- reduced and CNG-PM increases intracellular ROS production in iMGLs. **a** Representative green fluorescent and phase contrast images showing green signal in iMGLs at 3 h timepoint after adding the Cellrox reagent for cells that were pre-treated with vehicle, 50 µg ml^−1^ EN590-PM or CNG-PM either for 2 h or 24 h. Scale bars 400 µm. **b** Time curve for the count of green fluorescent Cellrox positive cell per cell density over 6-h live-cell imaging after 2-h exposure normalized to maximum value of vehicle. **c** Time curve after 24-h pretreatment. **d** Bar graphs after 2-h exposure from 4 separate iMGL batches comparing vehicle and CNG-PM groups. **e** bar graphs for 24-h pre-treatment comparing vehicle and EN590 groups. Data as mean ± SEM. Student t test, done to each batch separately. **p < 0.01, ***p < 0.001; n = 3 independent experiments each with 3–6 wells. Cell lines Bioni, MBE, MAD

While ROS are an important part of the arsenal employed by microglia in tissue defense, unbalanced ROS production contributes to oxidative stress and is hypothesized to lead to neuronal death in neurodegenerative diseases [48]. The cells were exposed to PMs for 2 and 24 h before fluorescent Cellrox reagent was added, and fluorescence was measured continuously by live-cell imaging for 6 h. For 2 h exposure, the analysis was done 2 h and for 24-h exposure 3.5 h after addition of Cellrox, when a clear distinction in green fluorescent objects was detected between vehicle and PM groups (Fig. 6a). Time curves for Cellrox green object count per cell density after the 2-h pre-treatment revealed different outcomes for CNG-PM compared to vehicle exposure (Fig. 6b). Indeed, quantification at the 2-h timepoint showed that 50 µg ml^−1^ CNG-PM increased ROS (*p* < 0.001) significantly (Fig. 6d) in four separate iMGL batches. EN590-PM did not cause significant changes after 2 h of exposures. Instead, after 24-h pretreatment with EN590-PM, there was a significant reduction in ROS production at 3.5-h timepoint (*p* < 0.001), whereas CNG-PM did not differ from vehicle treatment at this exposure paradigm (Fig. 6d, e).

### CNG-PM induces secretion of distinct cytokines and chemokines compared to LPS in human iMGLs

**Fig. 7:**
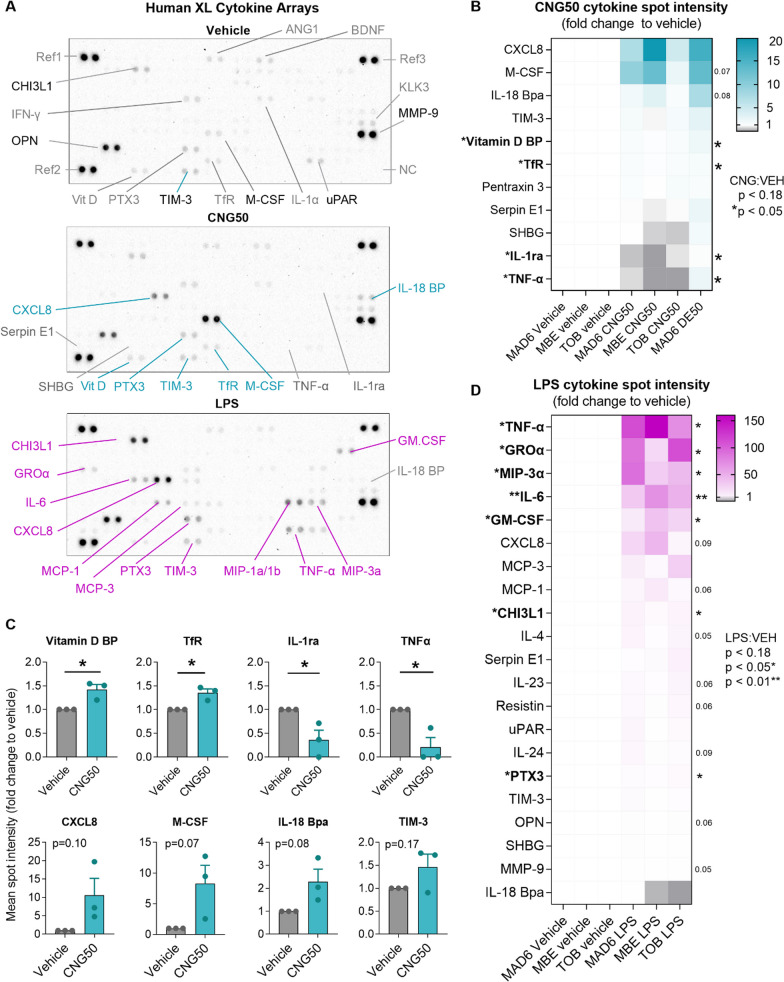
CNG-PM induces secretion of distinct cytokines compared to LPS in human iMGLs. Cytokine production was evaluated using proteome profiler arrays. Cells were stimulated for 24 h, and supernatants were harvested, pooled from three technical replicates and used for experiments. **a** Representative dot plots for vehicle, CNG50 and LPS 20 ng ml^−1^ cytokine arrays. **b** Fold change in mean spot intensity of CNG50 compared to vehicle represented as a heat map for analytes with *p* < 0.18. Turquoise upregulated cytokines; white unchanged; grey downregulated cytokines. **c** Respective fold change in mean spot intensity of LPS compared to vehicle represented as a heat map for analytes with *p* < 0.18. **d** Quantification of heatmap data presented as bar graphs. Data as mean ± SEM. Non-parametric Kruskal–Wallis test followed by Dunn’s test. *p < 0.05, **< 0.01, ***p < 0.001. n = 3 biological replicates each containing 3 pooled technical replicates. See also Figs. 8 and 9

**Fig. 8:**
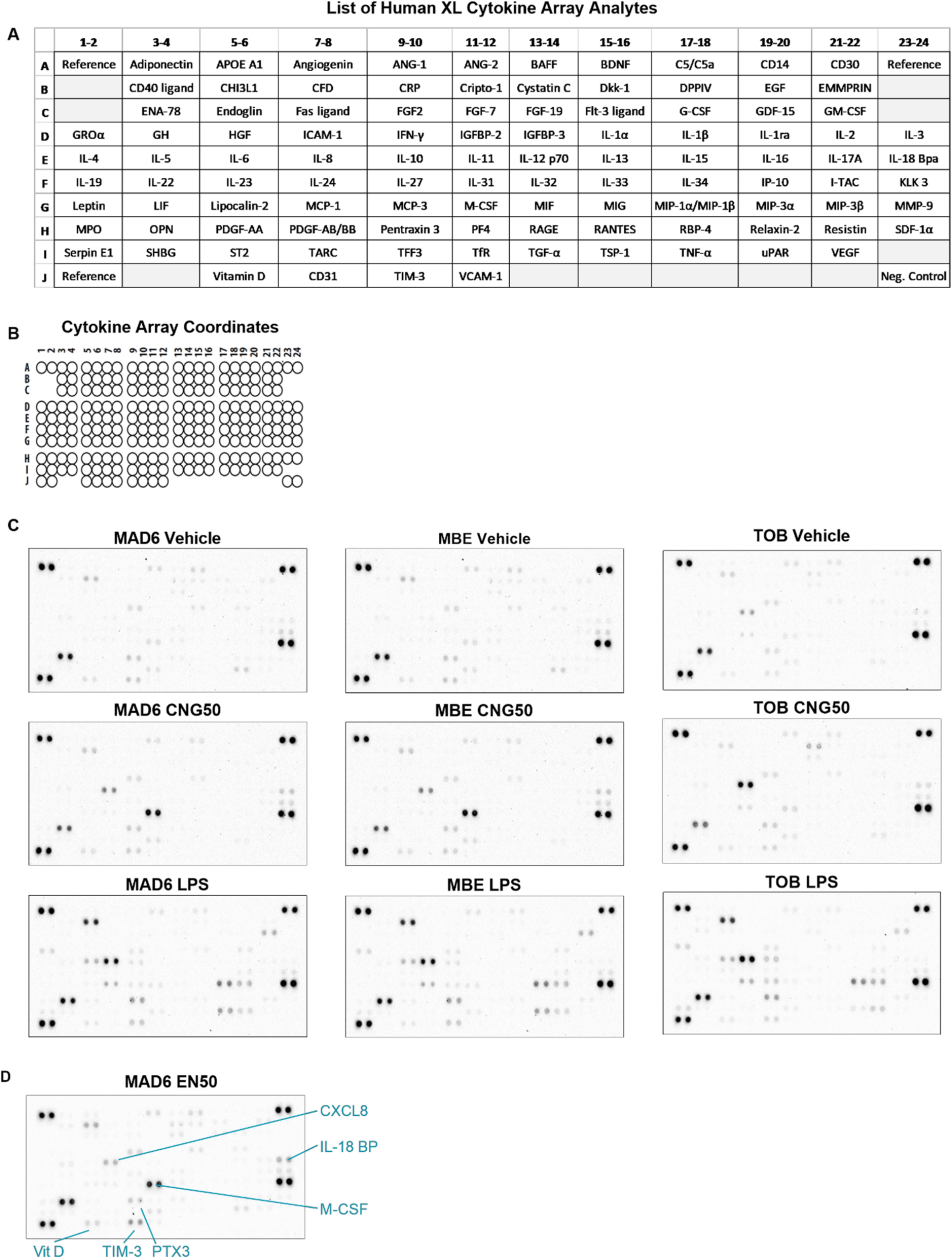
Human cytokine arrays used for producing Fig. 7 data and graphs. **a** List of analytes, **b** coordinates and dot plots **c** for 24 h treated vehicle, CNG50 and LPS 20 samples for three biological replicates (MAD6, MBE2968, and TOB cell lines) each containing 3 pooled technical replicates. **d** A dot plot for EN50 treated samples

**Fig. 9:**
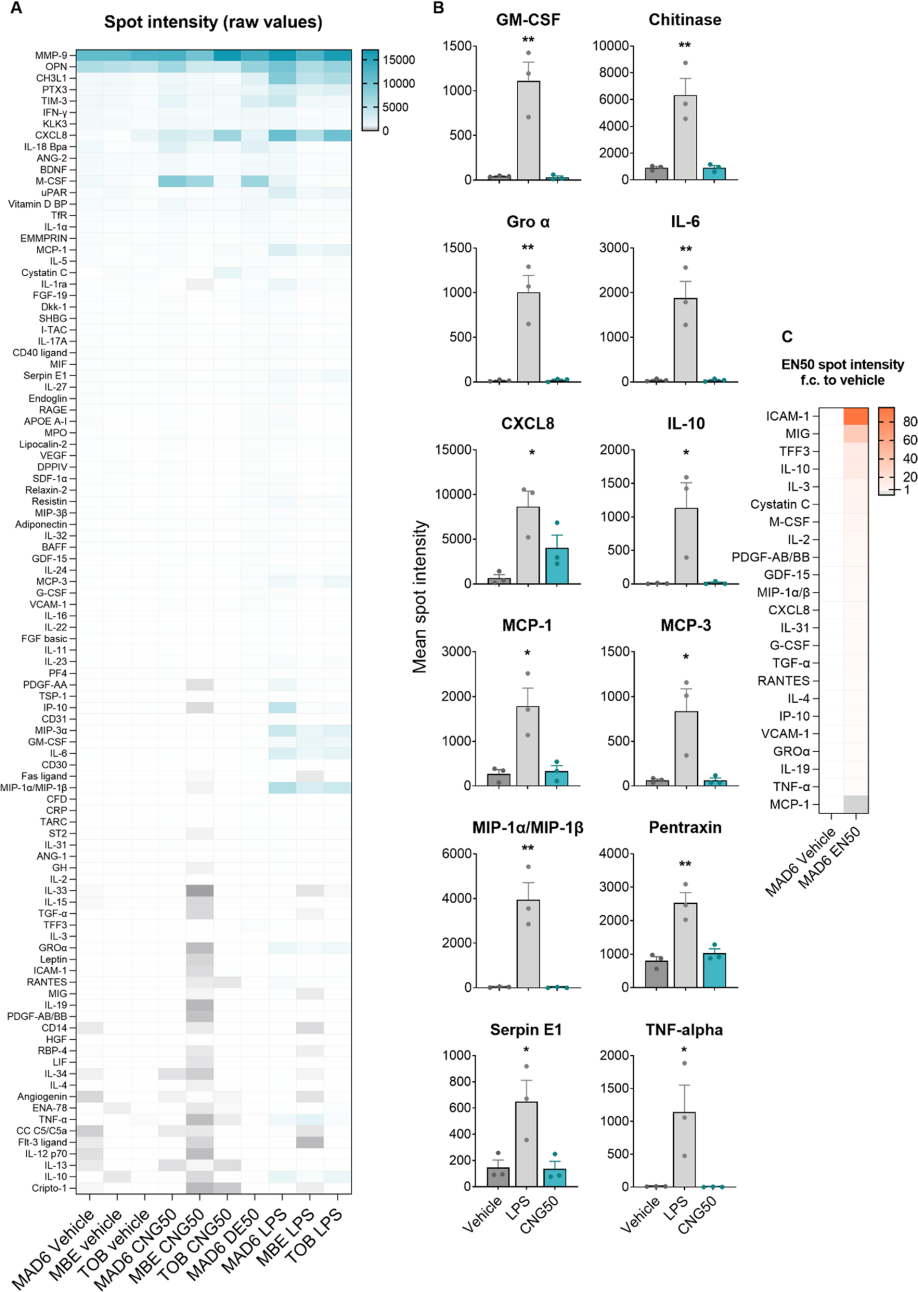
Cytokine proteome profiler arrays of secreted cytokines in human iMGLs. Cells were stimulated for 24 h, and supernatants were harvested, pooled from three technical replicates and used for experiments. **a** Raw values for spot intensity in all analyzed samples presented as a heatmap after normalization to negative control and reference spots of each plot. Turquoise highly detected cytokines; white middle; grey lowly detected cytokines. **b** Quantification of heatmap data for vehicle, LPS and CNG50 presented as bar graphs. n = 3 biological replicates each containing 3 pooled technical replicates. **c** Respective fold change heatmap for mean spot intensity of EN50 (n = 1 array) compared to vehicle represented for 24 analytes with biggest differences. Data as mean ± SEM. Non-parametric Kruskal–Wallis test followed by Dunn’s test. *p < 0.05, **< 0.01, ***p < 0.001

We next explored whether EN590-PM or CNG-PM alter microglial cytokine secretion by using a proteome profiler for 105 cytokines, chemokines, and acute phase proteins after the 24 h exposure to vehicle, 50 µg ml^−1^ EN590-PM or CNG-PM. 20 ng ml^−1^ LPS was used as a positive control to induce proinflammatory cytokine secretion (Figs. 7a and 8). Dot plots revealed that vehicle-treated iMGLs secreted measurable amounts of chitinase 3-like 1 (CHI3L1), osteopontin 1 (OPN1/SPP1), metalloproteinase 9 (MMP-9), Urokinase Plasminogen Activator Surface Receptor (uPAR) and T Cell Immunoglobulin Mucin 3 (TIM-3) (Figs. 7a and 8). Several analytes had dim signals in basal media, either indicating the presence of these analytes in the media or unspecific signal. Significant 1.5-fold increase in Vitamin D binding protein (Vit D) and transferring receptor-1 (Tfr) secretion were observed in CNG50 treated iMGLs compared to vehicle (Fig. 7b, d). In contrast, CNG50 induced 0.5-fold decrease in the secretion of IL-1ra and TNFa compared to vehicle (Fig. 7b, d). Larger differences in mean dot plot intensity between CNG50 and vehicle were observed in CXCL8, M-CSF and IL-18 BP, which were more intense in CNG50 plots (Figs. 7a, b and 8), but did not reach statistical significance upon quantification (Fig. 7d). LPS plots and their quantification demonstrated significant increases in larger range of analytes compared to vehicle (Figs. 7c, 8 and 9). Among them, GM-CSF, IL-6, CXCL8, MCP-1 and TNFa were in line with another study using the same iMGL differentiation protocol and LPS stimulus but different cytokine analysis assay [39]. For EN590, there was only one biological replicate and thus it was not possible to perform significance analysis (Fig. 8). However, comparison of the mean dot intensity to vehicle indicated similar a trend for increased CXCL8, M-CSF and IL-18 BP and possibly also for vitamin D binding protein, TIM-3 and Pentraxin 3 (PTX3) as was demonstrated for CNG50 with three biological replicates (Figs. 8 and 9).

## Discussion

Traffic related PM has become a global health concern upon increased incidences of adverse health effects, including neurological disorders, in heavily trafficked areas [49–51]. PM from diesel exhaust is especially concerning as it can penetrate into the brain and absorb chemicals including PAHs, which exacerbate inflammatory responses [52, 53]. Compressed natural gas is widely concerned as an environmentally friendly alternative for diesel, since it shows low emission, high efficiency and clean combustion [54]. However, little is known about how these two PMs affect human microglia.

Understanding the impact of PM specifically on human microglia is important, since human microglial functions, transcriptional signatures and regulation have been shown to be fundamentally different from their rodent counterparts [34, 35, 55–57]. Here we generated human iMGLs from four donor iPSC cell lines and examined the impact of PM exposure on cell survival, metabolism, phagocytosis, and ROS and cytokine production. Our data demonstrate that even a short 24-h exposure to CNG-PM and EN590-PM alter the key functions of human iMGLs. The concentrations and exposure times used in this study were based on earlier in vitro primary rodent and immortalized microglial cell line studies using 1–500 ug ml^−1^ concentrations for short 24–48 h [21, 36, 58]. While we lack comprehensive knowledge of PM amounts and bioavailability in the brain, emerging studies indicate that PM can passage into the brain [21, 59–62]. Evidence of PM’s existence in the brain was recently obtained in a publication measuring magnetized nanoparticles, with concentrations ranging from 0.2 to 12 µg g^−1^ in dry frontal cortex tissue samples of residents in Metropolitan Mexico City (MMC) and Manchester [63]. While PM concentrations remain elusive and cell culture and human brain are not fully comparable, this study seeks to gain a basal understanding whether PM impacts human microglia using sledgehammer high concentrations based on literature. To gain a better understanding of chronic low-level exposures modeling better the situation in individuals living in high-traffic areas, new long-term human stem cell derived culture models, such as brain organoids with iMGLs, should be used in future studies.

We observed a decrease in microglial death and increase in metabolism upon exposure to 10–300 µg ml^−1^ CNG-PM and 10 to 100 µg ml^−1^ EN590-PM compared to vehicle control by using three different viability assays. Moreover, we have used three different assays with confluency analysis and all indicate similar result, thus we consider the interpretation of these results robust. It is likely, that PMs activated a defense response in iMGLs and thus increased their viability compared to unstimulated control cells. On the contrary, Jalava and Anwar described a dose-dependent increase in cytotoxicity of the same pollutants and did not find increased cell survival of macrophages or kidney cells [37, 64]. This may be a cell-type or species-specific effect as Jalava and colleagues used the mouse macrophage cell-line RAW 264.7 and Anwar and colleagues used hamster kidney cells BHK-21 [37, 64]. Another explanation for the different responses between the studies could be the cell culture conditions. iMGLs were supplied daily with fresh nutrients and growth factors, including MCSF and IL-34, shown to support microglial survival and their physiological functions.

EN590 induced appearance of clearly visible particles in the treated cell cultures, whereas these particles were not observed in CNG cultures. This is in line with the higher content of carbon black, the main constituent of visible particles, in diesel exhaust compared to CNG [38]. EN590 treated iMGLs exhibited also elongated morphology likely reflecting their migration and clustering around the EN590 particles, whereas CNG treated cells had a similar amoeboid morphology as vehicle DMSO treated cells. Both pollutants were found to equally reduce phagocytosis of zymosan bioparticles in line with the data obtained in animal models [36]. It is likely, that iMGLs phagocytosed PMs during the 24 h pretreatment period and when the bioparticles were introduced during the assay, the phagolysosomal system of iMGLs was already saturated by PMs. In line with our results, rodent microglia typically show changes in morphology, production of inflammatory cytokines, a metabolic shift and phagocytosis resulting in oxidative stress [36]. On the other hand, data on pollutant exposed human neuron, astrocyte and microglia triculture support our observation of decreased ROS production and increased TNF-α secretion upon CNG-PM treatment [32]. Characterization of CNG-PM suggests that these morphological differences likely reflected the physical and chemical content of the PM [65]. The aerodynamic diameter of EN590 PM was reported to be between 30 and 70 nm, while the number of measurable particles in CNG exhaust was too small for size distribution analysis [37, 38]. It is likely that the mass of CNG-PM contains smaller ultra-fine particles that were not detected by the technology used at the time of characterizing the PMs. Importantly, exposures in this study were not reflecting only UFP effects as the filters collect also larger particles. Furthermore, large aggerates in EN590-PM fractions may hamper comparison of the pollutants while some of PM constituents are unavailable in solution when they are stuck in agglomerates. Differential effects reported for EN590-PM and CNG-PM could therefore be an artefact from comparing particle aggregates of massively different size.

EN590-PM contains a tenfold higher concentration of PAHs compared to CNG [37]. PAHs are a class of organic compounds produced by incomplete combustion and often consist of three or more fused benzene rings containing only carbon and hydrogen. PAHs are relatively insoluble in water, and they can bind to or form small particles in the air. This organic fraction also most probably caused the formation of aggregates in diesel exhaust samples visible in bright-field images. CNG-PM contains a higher amount of inorganic salts (NO_3_^−^, Na^+^, SO_4_^2−^, NH_4_^+^, Cl^−^, K^+^) compared to EN590. The inorganic salts principally maintain the osmotic balance in the cell culture media and may help to regulate membrane potential through the ions required in the cell matrix for cell attachment and as enzyme cofactors. While the osmolarity was not measured in this study, live imaging showed stronger attachment and survival of iMGLs in response to both EN590 and CNG compared to the vehicle over time. Although investigation of the exact PM constituents responsible for the differences between the observed effects was out of the scope of the current study, our data suggest that different vehicle exhaust related PMs can elicit distinct functional changes in human microglia.

In general, neither of the pollutants were toxic for the iMGLs in our experiments. Unfortunately, due to poor availability of the pollutants, we could not repeat these experiments three times and thus, we did not see any significant differences. CNG exhaust is considered to be less harmful than diesel emissions. However, opposite results have been described for macrophages [37] and other cell types [64, 66]. Higher cytotoxicity of CNG-PM compared to EN590 could stem from matching the PM weight in the samples and concentrating CNG-PM more as it naturally has smaller PM content compared to EN590 exhaust, thus resulting in the high number of heavy metals in CNG. While both PMs contain relatively high concentrations of the essential heavy metal Zn, all other heavy metals were higher in CNG compared to EN590, including essential heavy metals Fe, Cu, Mn, Cr, Co, and Ni and non-essential poisonous metals Pb, V, and Cd. Metal overload toxicity in neurological disorders by redox mechanisms has been well-documented [67] and is extensively reviewed in regards to cognitive dysfunction in neurodegenerative diseases [68–70]. These metals are present in cell culture medium as bi-or trivalent cations that can enter the cells using channels that are shared with iron or calcium [65, 71]. Our protein array showed increased levels of transferrin and vitamin D binding proteins in cell culture media upon CNG-PM exposure, further supporting heavy metal mediated effects, since transferrin mobilizes, and vitamin D stimulates the absorption of heavy metals [72–74]. Thus, excessive vitamin D binding protein and transferrin produced by microglia in response to PM exposure may lead to increased metal absorption and retention in the brain, thus contributing to brain pathologies. However, the observed differences in fold changes in secreted cytokines are rather small (+ 1.5–0.5) and could be reflecting the overall cell health and viability. Thus, their biological importance should be confirmed with further studies.

## Conclusions

Here, we demonstrate that human microglia exhibit robust and differential functional responses to EN590 and CNG. The concentrations used were higher than the ones reaching the brain and thus, further studies with more complex multicellular systems and chronic and lower exposure concentrations are needed. They will hopefully reveal mechanistic insights possibly providing means for mitigation of the harmful air pollution induced effects. A better understanding of the adverse effects of exhaust may result in a widely used enhanced diesel alternative, with both environmental and health effects considered.

## Methods

### The description of human iPSC lines

The origin and characteristics of the four human iPSC lines used in this study are described in Table 2. All iPSC lines were previously generated from skin biopsies obtained from three healthy male and one healthy female donor at ages between 15 and 73 years and were characterized to have a normal karyotype, pluripotency and they were confirmed to be free of pathogens [39, 40, 45–47]. All experiments with human iPSC derived cells were performed in accordance with the Declaration of Helsinki and were approved by the Research Ethics Committee of the Northern Savo Hospital District (license no. 123/2016). The commercial BIONi010‐C-2 line was purchased from the European bank for Induced Pluripotent Stem cells (EBiSC). It was genetically edited by the producer (Copenhagen, Denmark) to have two APOE3/3 alleles and was reported to carry a nonsignificant duplication of 1.4 Mbp on Chr22 in q11.23.

### Traffic-related particulate matter

Traffic-related PM was collected at VTT Technical Research Centre of Finland Ltd. in an engine laboratory and analyzed and described by Jalava et al. [37, 38]. Briefly, EN590-PM was collected from exhaust gas of conventional EN590 diesel fuel with low sulfur (8 mg kg^−1^) and PAH (1%) contents combusted in a heavy-duty direct injection, turbocharged and intercooled Scania 2005 engine attached to an engine dynamometer. The six-cylinder 11.7-L engine with maximum power of 310 kW and torque of 2100 Nm fulfilled the EURO IV emission class requirements and had exhaust gas recirculation system. CNG-PM was collected from exhaust of compressed natural gas (NGS) combusted through a stoichiometric 11.9-L six-cylinder engine, driven on a chassis dynamometer, on a 2008-year model commuter bus with 206,000 driven kilometers. The CNG bus filled the requirements for the EEV (Enhanced environmentally friendly vehicle) emission class, in which the particle mass emissions are between Euro V and VI standards. Both the diesel engine and the CNG bus were operated using a Braunschweig cycle that reflects the driving conditions of the urban commuter traffic buses with multiple stops and low load conditions. The PM samples were collected on polytetrafluoroethylene (PTFE; Fluoropore FSLW14200, 142 mm, Millipore) filters from a constant volume dilution tunnel using a high-volume sampler (800 L per minute). Blank filter controls were treated similarly without the exhaust gas. The filters were weighted before and after the collection to obtain mass for the collected PM. Filters were cut into pieces and PM was extracted into methanol in glass tubes with 2 × 30 min ultrasonication. The methanol extracts were dried in glass tubes under 99.5% nitrogen gas flow and stored at − 20 °C. PM was characterized for the chemical and particle composition (Table 1) as published earlier [37]. The particulate mass results for EN590 and CNG PM used in this study were reported earlier [35, 62]. Particle number size distributions were measured with the Electrical Low Pressure Impactor (ELPI) instrument (Dekati Ltd) and the total number of particles was determined with Condensation Particle Counter (CPC, TSI model CPC 3022A). The aerodynamic diamater of EN590 PM was reported to be between 30 and 70 nm with a low contribution of particles in the size class 100–300 nm, while the number of measurable particles in CNG exhaust was too small for size distribution analysis [35, 62]. The sampling was done from the dilution tunnel without any size selection, thus including both ultra-fine and more coarse particles Prior to the cell culture experiments, PM was dissolved in 100% dimethyl sulfoxide (DMSO; D2650, Sigma) and diluted in sterile H_2_O (KK7119, Baxter) to 1 mg ml^−1^ stock in final 10% DMSO. The solution was sonicated for 30 min in the glass tubes, aliquoted into Eppendorf tubes (FB74111, Fiskerbrand) and stored at − 20 °C maximum of 2 months before further use. Both CNG and EN590 samples were processed similarly to avoid processing bias.

### Human iPSC maintenance

iPSCs were maintained in essential 8 (E8) medium (A151700, Gibco) supplemented with 0.5% penicillin/streptomycin (P/S, 15140122, Invitrogen) on growth factor reduced Matrigel™ (356231, Corning) coated 3.5 cm dishes (83 3900, Sarstedt). Small colonies were passaged twice a week with 0.5 mM EDTA (15575, Gibco) in the presence of 5 µM rho kinase (ROCK) inhibitor (S1049, Selleckchem). Up to 20 passages were used and cultures were tested regularly to be negative for mycoplasma (LT07, Lonza).

### Differentiation of iMGLs

iPSCs were differentiated into iMGLs as previously described [39]. On differentiation day D16, iMGLs were seeded in microglia medium containing IMDM (21980032, Gibco™), 10% [v/v] heat-inactivated hiFBS (10500, Gibco™), 0.5% [v/v] P/S, 10 ng ml^−1^ MCSF (300–25, Peprotech) and 10 ng ml^−1^ IL-34 (200-34, Peprotech) on PDL (P6407, Sigma) coated vessels with desired cell densities for experiments: 47.000 cells per cm^2^ (15,000 cells per well) on 96-well Nunclon plates (167008, Thermo Fisher) for phagocytosis, Cellrox, Cytotox, MTT, LDH and cytokine experiments or 94.000 cells per cm^2^ (30,000 cells per well) on µ-Slide 8-Well plates (80826, Ibidi) and immunofluorescence stainings. Half of the medium was changed every 24 h for 5–9 days until starting the experiments on *D*21 to *D*25 when cells were treated according to desired assays.

#### Cytotox Green assay

The survival of iMGLs was assessed using the Cytotox™ Green reagent (4633, Essen Bioscience) and Incucyte™ S3 Live-Cell Analysis System (4647, Essen Bioscience). Prior to carrying out the reported data, we performed pilot experiments to demonstrate that PM samples did not affect the used assays by using particles only without any cells as a control for fluorescent incucyte assays and for MTT and LDH assays. In these pilot experiments we detected no effect of PM indicating that these pollutants do not react with MTT, LDH or cytotox green reagents. For the incucyte experiments presented in this study, we also carried out initial tests where half of the plate was treated with PMs and Cytotox while other half of the plate was treated only with PMs (no Cytotox). Analyzing confluency of exposed cells with or without cytotox on the same plate goes hand in hand with our MTT, LDH and Cytotox green results. In the beginning of the assay, cells on 96-well plates were treated with 10, 25, 50, 100, 200 or 300 µg ml^−1^ EN590- or CNG-PM in microglia medium excluded of hiFBS and supplemented with 250 nM Cytotox™ Green reagent. As positive controls, 400 µM 1-methyl-4-phenylpyridinium (MPP+; D048, Sigma Aldrich) or 40% DMSO were used for killing the cells over the experiment time. Two images per well were taken to image cells with phase contrast and green fluorescent channels at 20× magnification every 3 h for a total of 60 h to record cell density and the number of Cytotox™ green positive dead cells. Images were quantified using Incucyte Software; fluorescence intensity of Cytotox green reagent was separated from the auto-fluorescent effects of EN590- and CNG-PM and background fluorescence using adaptive segmentation. Mask of fluorescent nuclei above the threshold were created and quantified as object counts that were normalized to cell density.

### LDH assay

CyQUANT™ LDH Cytotoxicity Assay Kit (C20301, Invitrogen) was used according to manufacturer’s instructions for measuring lactate dehydrogenase (LDH) enzyme in the cell culture media as indication of compromised cell membrane aka cytotoxicity. The cells were seeded and treated similarly as for Cytotox green assay, and the medium was collected after 24 h. In the assay, the extracellular LDH catalyses the conversion of lactate to pyruvate via NAD+ reduction to NADH accompanied with a red formazan formation proportional to the amount of LDH. The absorbances at 490 nm and 680 nm were measured using Victor Wallace plate reader and LDH activity determined by subtracting the background absorbance and then normalizing to the positive control or maximum LDH value elicited by lysing the cells prior to the assay. The intensity of the colored product is directly proportional to the number of viable cells.

### MTT assay

The colorimetric MTT assay was used to measure cellular metabolic activity as an indicator of cell viability and cytotoxicity. The cells were seeded and treated for 24 h similarly as for LDH assay. After the incubation period, 2% Triton X-100 was added for 5 min to negative control wells to kill the cells. Then the yellow tetrazolium salt (3-(4,5-dimethylthiazol-2-yl)-2,5-diphenyltetrazolium bromide) or MTT labeling reagent (Sigma) was added at a final concentration of 0.5 mg ml^−1^ to each well and incubated for 4 h in an incubator at 37 °C, 5% CO_2_ to let the cells metabolize the MTT into purple formazan crystals that were detected under microscope. The viable cells contain NAD(P)H-dependent oxidoreductase enzymes which reduce the MTT to formazan. Medium was removed and the cells and formazan were solubilized completely in DMSO overnight. The absorbances were measured at 550 nm using a Victor Wallace plate reader and metabolic activity was determined by normalizing to negative control wells that got a value of zero. The intensity of the colored product is directly proportional to the number of viable cells.

### Phagocytosis

The phagocytic capacity of iMGLs was quantified using green pHrodo™ Zymosan A bioparticles™ (P35365, Invitrogen) with Incucyte™ live-cell imaging on 96-well plates. Cells were pre-treated for 24 h with 10, 50 and 100 µg ml^−1^ EN590- or CNG-PM in microglia medium not containing hiFBS. After 24 h, medium was replaced by Optimem medium (31905847, Gibco) containing EN590- or CNG-PM and 133 µg ml^−1^ pHrodo™ Zymosan A bioparticles™. Cells were imaged with phase contrast and green fluorescent channels at 20× magnification with two images taken per well every 30 min for 6 h to record increased fluorescence of pHrodo™ Green conjugates at acidic pH in phagosomes over time. Using Incucyte Software, the integrated fluorescence intensity was quantified after excluding background fluorescence with adaptive segmentation thresholding and was normalized to cell density prior to the addition of pHrodo Bioparticles.

### Reactive oxygen species production

The intracellular accumulation of reactive oxygen species (ROS) was assessed using Cellrox Green reagent (C10444, Invitrogen) and Incucyte live-cell imaging with 96-well plates. Cells were pre-treated with 10, 50 and 100 µg ml^−1^ EN590- or CNG-PM in microglia medium without hiFBS for two or 24 h. After the preincubation time, medium was replaced by medium containing EN590- or CNG-PM and 3 mM Cellrox Green. Cells were imaged with phase contract and a green fluorescent channel at 20× magnification with four images taken per well every 30 min for 6 h. Count of Cellrox green positive cells over time were analyzed and normalized to cell density similarly as for Cytotox green assay at 3-h timepoint.

### Cytokine secretion profiler

A membrane-based sandwich immunoassay, The Proteome Profiler Human XL Cytokine Array Kit (ARY022B, R&D Systems), was used for detecting 105 cytokines, chemokines, and acute phase proteins in the supernatants collected from 96 well plates. After the 24-h exposure to vehicle, 50 µg ml^−1^ EN590-PM, 50 µg ml^−1^ CNG-PM or 20 ng ml^−1^ LPS O111:B4 in serum-free media, 100 ul medium from 3 technical replicates was collected and pooled to get one sample. This was repeated with 3 different cell lines (n = 3 biological replicates for CNG-PM. n = 1 for EN590-PM). LPS (n = 3) was used as a positive control to induce cytokine secretion. Media were kept on ice, centrifuged at 300×*g* for 5 min and stored at − 70 °C until the analysis according to the manufacturer’s instructions. Briefly, cytokine membranes were blocked, after which they were incubated with the samples over night at 4 °C. Next day, the membranes were washed and incubated for 1 h with the detection antibody and 30 min with Streptavidin-HRP, after which they were imaged using Bio Rad ChemiDoc MP device (Bio Rad). Profiles of mean spot pixel density were quantified using Image Lab 5.1 program (Bio Rad). Spots were circled using same sized ROI for all the membranes. Negative control spot values were subtracted as background and the results were normalized for reference spots of each membrane. Mean intensity of each spot was used for the analysis. The results are represented as fold change to the vehicle group of each cell line.

### Immunocytochemistry and confocal microscopy

At *D24,* iMGLs on PDL-coated 8-well plates (80826, Ibidi GmbH) were fixed in + 37 °C pre-warmed 4% paraformaldehyde (PFA) for 20 min, washed with DPBS and stored at 4 °C. Cells were permeabilized in 0.5% Tween (93773, Sigma), 0.2% Triton (9002931, Sigma) and 5% normal goat serum (NGS) for 20 min. Non-specific binding was blocked with 0.2% Triton in 10% NGS for 2 h. Primary antibodies for triggering receptor expressed on myeloid cells 2 (TREM2), a purinergic receptor (P2RY12), a transmembrane receptor (TMEM119), a transcription factor (PU1), chemokine receptor 1 (CX3CR1) and transcription growth factor beta receptor 1 (TGFbR1) (Table 3) were incubated overnight in 0.2% Triton and 5% NGS at 4 °C. Cells were washed with PBS and incubated overnight with a fluorophore containing secondary antibody Alexa Fluor 568 (AF568) goat-anti-rabbit (Table 3) at 4 °C. Cells were washed with PBS and nuclei were stained with 2.5 mg ml^−1^ bisbenzimide H33342 trihydrochloride (DAPI; B2261, Sigma-Aldrich). Images were captured using a Zeiss LSM 800 Airyscan confocal microscope (Zeiss Germany). Two randomly selected images were taken per well at 60× and 40× magnification. Red fluorescent signal was captured with a laser at 568 nm and a blue fluorescence at 405 nm. Images were processed by adjusting the brightness and contrast and exported using FIJI software (ImageJ).

**Table 3:** Antibodies used for immunostaining

Specificity	#	Source	Concentration
Trem2	91068S	New England biolabs	1:200
P2RY12	HPA14518	Sigma	1:125
Tmem119	ab185333	Abcam	1:100
PU1	2266S	Cell signal	1:200
TGFbR1	ab31013	Abcam	1:200
AF568 goat-anti-rabbit	A-11011	Thermo fisher	1:500

### Statistical analysis

All experiments were performed in triplicate or sextuplicate wells and repeated two to three times using different biological cell lines in independent experiments (Table 2). If the batches were combined (cytotoxicity and cytokine array), the data was not normally distributed and Kruskal–Wallis test was used to compare the mean rank to vehicle. Multiple comparisons were corrected using Dunn’s statistical hypothesis testing and confidence level 0.05 is reported. Significance was reported only for those groups that had data from three independent batches/experiments. If batches were analysed separately, One-way ANOVA or student’s t test were used. All groups were normalized and compared to the vehicle condition. Incucyte values were not included when they were found to be significant outliers or images showed abnormalities. Statistical analysis was done, and graphs were made using GraphPad Prism 9 (GraphPad Software). *p* values below an α of 0.05 were considered significant.

### Supplementary Information

**Additional file 1: Video S1.** Timelapse video with 30-min interval and over 5.5 h period after treatment with 50 ug ml^−1^ EN590 depicting iMGLs clustering around and interacting with the air pollutant particles.

**Additional file 2: Video S2.** Timelapse video with 30-min interval and over 5.5 h period after treatment with vehicle depicting iMGLs surveying their proximal environment but not moving further away.

## Data Availability

The datasets used and/or analyzed during the current study are available from the corresponding author on reasonable request.

## Abbreviations

Aβ: Amyloid beta
AD: Alzheimer’s disease
ADP: Adenosine diphosphate
AF: Alexa fluor
APOE: Apolipoprotein
ATP: Adenosine triphosphate
APP: Apolipoprotein precursor protein
AUC: Area under curve
BBB: Blood–brain barrier
BMP4: Bone morphogenic factor 4
CNG: Compressed natural gas
CNS: Central nervous system
COPD: Chronic obstructive pulmonary disorder
CRISPR: Clustered regularly interspaced short palindromic repeats
CX3CR1: Chemokine receptor 1
DAPI: Bisbenzimide H33342 trihydrochloride
DE: Diesel exhaust
DMSO: Dimethyl sulfoxide
DNA: Deoxyribonucleic acid
DPBS: Dulbecco’s phosphate buffered saline
EDTA: Ethylenediaminetetraacetic acid
EN590: A conventional diesel fuel: meeting European standard
E8: Essential 8
FGF: Fibroblast growth factor
hiPSCs: Human induced pluripotent stem cells
HPC: Hematopoietic progenitor cells
hiFBS: Heat-inactivated fetal bovine serum
IGF: Insulin-like growth factor 2
IL: Interleukin
LDH: Lactate dehydrogenase
LPS: Lipopolysaccharide
LOAD: Late onset Alzheimer’s disease
iMGs: Induced microglial-like cells
MCSF: Macrophage colony stimulating factor
MPP: 1-Methyl-4-phenylpyridinium
MTT: Metabolic activity assay
NFT: Neurofibrillary tangles
NGS: Normal goat serum
PDL: Poly-d-lysine
PM: Particulate matter
P/S: Penicillin/streptomycin
P2RY12: Purinergic receptor P2Y12
qRT-PCR: Quantitative reverse transcriptase polymerase chain reaction
ROCK: Rho kinase
ROS: Reactive oxygen species
SCF: SKP1-cullin-F-box protein
TBHP: Tert-butyl hydroperoxide
TGFbR1: Transcription growth factor beta receptor 1
TMEM119: Transmembrane protein 119
TPO: Thrombopoietin
TRAP: Traffic-related particulate matter
TREM2: Triggering receptor expressed on myeloid cells 2
UFP: Ultra-fine particles
ULA: Ultra-low attachment
VEGF: Vascular endothelial growth factor
